# Crystal structure of an unknown solvate of bis­(tetra-*n*-butyl­ammonium) [*N*,*N*′-(4-tri­fluoro­methyl-1,2-phenyl­ene)bis­(oxamato)-κ^4^ *O*,*N*,*N*′,*O*′]nickelate(II)

**DOI:** 10.1107/S205698901500835X

**Published:** 2015-05-07

**Authors:** François Eya’ane Meva, Dieter Schaarschmidt, Tobias Rüffer

**Affiliations:** a Department of Pharmaceutical Sciences, Faculty of Medicine and Pharmaceutical Sciences, University of Douala, BP 2701, Cameroon; b Technische Universität Chemnitz, Faculty of Natural Sciences, Institute of Chemistry, Inorganic Chemistry, 09107 Chemnitz, Germany

**Keywords:** crystal structure, nickel(II), oxamate ligand, non-symmetric compound, disorder, SQUEEZE procedure

## Abstract

In the title compound, [N(C_4_H_9_)_4_]_2_[Ni(C_11_H_3_F_3_N_2_O_6_)] or [N(*n*-Bu)_4_]_2_[Ni(topbo)] [*n*-Bu = *n*-butyl and topbo = 4-tri­fluoro­methyl-1,2-phenyl­enebis(oxamate)], the Ni^2+^ cation is coordinated by two deprotonated amido N atoms and two carboxyl­ate O atoms, setting up a slightly distorted square-planar coordination environment. The [Ni(topbo]^2−^ anion lies on a twofold rotation axis. Due to an incompatibility with the point-group symmetry of the complete mol­ecule, orientational disorder of the CF_3_ group is observed. The tetra­hedral ammonium cations and the anion are linked by weak inter­molecular C—H⋯O and C—H⋯F hydrogen-bonding inter­actions into a three-dimensional network. A region of electron density was treated with the SQUEEZE procedure in *PLATON* [Spek (2015). *Acta Cryst*. C**71**, 9–18] following unsuccessful attempts to model it as plausible solvent mol­ecule(s). The given chemical formula and other crystal data do not take into account the unknown solvent mol­ecule.

## Abstract 2

In the title compound, the Ni^2+^ cation is coordinated by two deprotonated amido N atoms and two carboxyl­ate O atoms, setting up a square-planar coordination environment. The cations and the anion are linked by weak intra- and inter­molecular C—H⋯O and C—H⋯F hydrogen bonds.

## Chemical context   

Oxamate-bridged polymetallic complexes are of inter­est in the discipline of supra­molecular magnetism as they exhibit diverse supra­molecular architectures and magnetic properties (Pardo *et al.*, 2008 ▸; Kahn, 1987 ▸, 2000 ▸) and have been synthesized by, for example, Ruiz *et al.* (1997*a* ▸,*b* ▸), Berg *et al.* (2002 ▸), Martín *et al.* (2002 ▸) and Ottenwaelder *et al.* (2005 ▸). Over the last decade, we have been inter­ested in the synthesis of bis­(oxamates) and bis­(oxamate) complexes (Rüffer *et al.*, 2007*a* ▸,*b* ▸, 2008 ▸, 2009 ▸; Eya’ane Meva *et al.*, 2012 ▸), as well as their deposition as thin films (Bräuer *et al.*, 2006 ▸, 2008 ▸, 2009 ▸). In order to optimize the deposition conditions and to increase the thin-film quality, the monometallic title compound, bis­(tetra-*n*-butyl­ammonium) [*N*,*N*′-(4-tri­fluoro­methyl-1,2-phenyl­ene)bis­(oxa­mato)-κ^4^ *O*,*N*,*N*′,*O*′]nickelate(II), (I), was prepared. The complex includes four sites of coordination and a CF_3_ group which provides a good solubility in organic solvents.
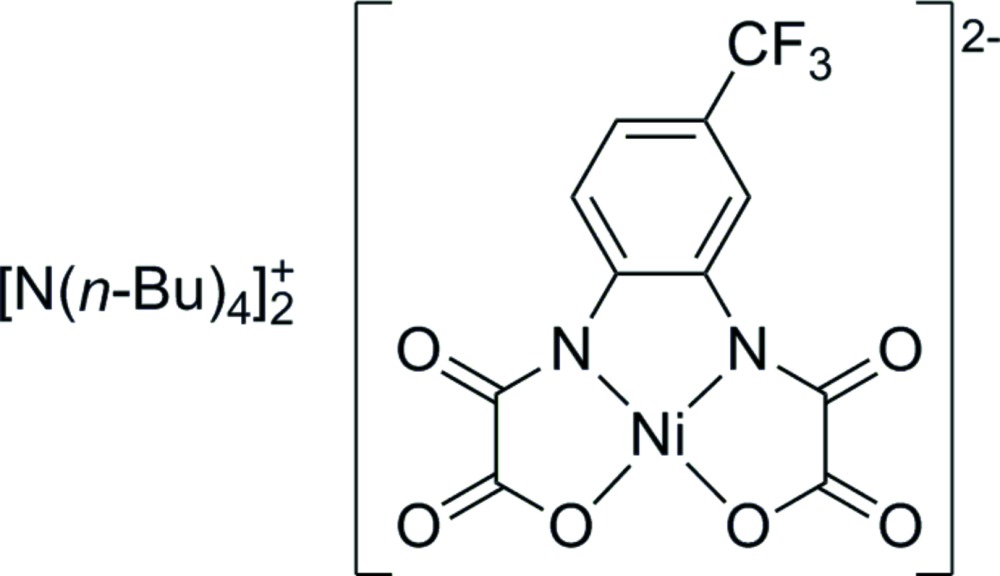


## Structural commentary   

**Figure 1:**
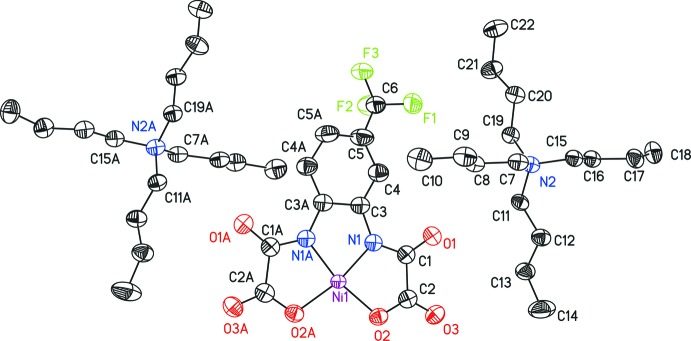
The mol­ecular components of (I) drawn with displacement ellipsoids at the 50% probability level. H atoms were omitted for clarity. Only one disordered part of the –CF_3_ group is shown. [Symmetry code: (A) −*x* + 2, *y*, −*z* + 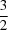.]

The asymmetric unit of compound (I) contains one [N(*n*-Bu)_4_]^+^ cation and half of the complex anion [Ni(topbo)]^2–^ (Fig. 1 ▸). The anion possesses point-group symmetry 2. This imposes orientational disorder of the CF_3_ group, which lies on both sides of the twofold rotation axis with 0.5 occupancy. The anion is essentially planar (root-mean-square deviation 0.145 Å), the highest deviation from planarity being observed for C6 [0.440 (5) Å]. The Ni^2+^ cation is coordinated by two deprotonated amido N atoms and two carboxyl­ate O atoms, resulting in a slightly distorted square-planar coordination geometry. In agreement with related nickel compounds, the Ni—N bonds are significantly shorter than the Ni—O bonds, which is due to the stronger donicity of the amido nitro­gens (Fettouhi *et al.*, 1996 ▸; Rüffer *et al.*, 2007*a* ▸,*b* ▸, 2008 ▸; Abdulmalic *et al.*, 2013 ▸; Milek *et al.*, 2013 ▸). Compared to the respective nickel complex without the CF_3_ group (Abdulmalic *et al.*, 2013 ▸), compound (I) exhibits longer Ni—N and Ni—O bonds. It is instructive to note that for other complexes, the presence of electron-withdrawing substituents at the benzene moiety, *e.g.* Cl, NO_2_, causes a shortening of the Ni—N and Ni—O bonds (Fettouhi *et al.*, 1996 ▸; Rüffer *et al.*, 2008 ▸).

## Supra­molecular features   

**Figure 2:**
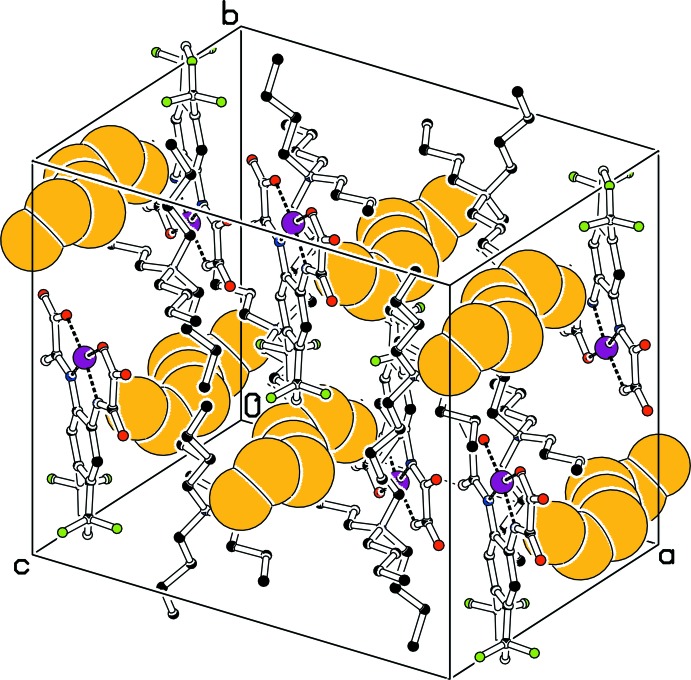
Packing diagram of compound (I), with voids in the structure represented by yellow spheres [drawn using the CAVITYPLOT routine in *PLATON* (Spek, 2009 ▸)]. H atoms are omitted for clarity. Color code: black (C), blue (N), red (O), green (F), purple (Ni).

Five weak C—H⋯O and one weak C—H⋯F hydrogen bonds (Steiner, 2002 ▸) are observed in the crystal structure of (I) (Table 1 ▸), which connect the [N(*n*-Bu)_4_]^+^ cations and the [Ni(topbo)]^2–^ anion, forming a three-dimensional network. A packing diagram is shown in Fig. 2 ▸.

## Synthesis and crystallization   

4-Tri­fluoro­methyl-1,2-phenyl­enebis(ethyl oxamate) was prepared from ethyl oxalyl chloride and 4-tri­fluoro­methyl-1,2-phenyl­enedi­amine in analogy to Cervera *et al.* (1998 ▸). To a solution of 4-tri­fluoro­methyl-1,2-phenyl­enedi­amine (0.4 g, 2.22 mmol) dissolved in tetra­hydro­furan (50 ml) was added dropwise *via* a dropping funnel a solution of ethyl oxalyl chloride (5.05 g, 4.45 mmol) in tetra­hydro­furan (25 ml) within 20 min. The resulting mixture was refluxed for 30 min at 343 K, filtrated and concentrated to about one third on a rotary evaporator. The careful addition of water resulted in the precipitation of a brown solid which was filtered off and dried in air.

To a solution of 4-tri­fluoro­methyl-1,2-phenyl­enebis(ethyl oxamate) (0.4 g, 1.06 mmol) in ethanol (40 ml) was added dropwise under stirring [N(*n*-Bu)_4_]OH (2.76 g, 4.25 mmol, 40 wt-% aqueous solution) in water (20 ml); the resulting mixture was refluxed for 30 min. After cooling to room temperature, an aqueous solution (20 ml) of NiCl_2_·6H_2_O (0.25 g, 1.05 mmol) was added dropwise under stirring. The yellow solution was filtered, concentrated to a volume of 20 ml on a rotatory evaporator, and extracted with di­chloro­methane (100 ml). The organic layer was separated, washed with water (3 x 25 ml) dried over Na_2_SO_4_ and concentrated to a volume of 10 ml. The title compound was precipitated by adding Et_2_O (100 ml). The yellow solid was filtered off, washed with Et_2_O and dried in air. Single crystals were obtained by the slow diffusion of Et_2_O into a saturated solution of the title compound in CH_2_Cl_2_/thf (1:1).

**Figure 3:**
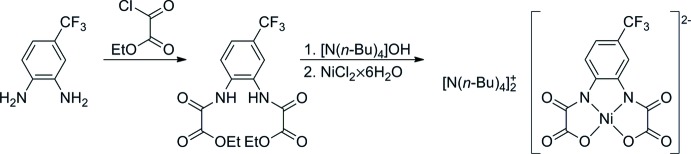
Scheme representing the synthesis of compound (I).

The overall synthetic procedure is schematically shown in Fig. 3 ▸.

## Refinement   

Crystal data, data collection and structure refinement details are summarized in Table 2 ▸. C-bonded H atoms were placed in calculated positions and constrained to ride on their parent atoms, with *U*_iso_(H) = 1.2*U*_eq_(C) and a C—H distance of 0.93 Å for aromatic and 0.97 Å for methyl­ene protons as well as *U*_iso_(H) = 1.5*U*_eq_(C) and a C—H distance of 0.96 Å for methyl protons.

A small region of electron density at a distance of 1.6–3.7 Å from the tri­fluoro­methyl group indicates the presence of a disordered solvent mol­ecule. All attempts to model a disordered tetra­hydro­furan, di­chloro­methane or diethyl ether mol­ecule (solvents used for crystallization) failed. Therefore, the solvent contributions have been removed using the SQUEEZE procedure in *PLATON* (Spek, 2015 ▸). SQUEEZE calculated a void volume of approximately 310 Å^3^ occupied by 24 electrons per unit cell. Fig. 2 ▸ shows the positions of the voids within the unit cell.

## Supplementary Material

Crystal structure: contains datablock(s) I, New_Global_Publ_Block. DOI: 10.1107/S205698901500835X/wm5144sup1.cif

Structure factors: contains datablock(s) I. DOI: 10.1107/S205698901500835X/wm5144Isup2.hkl

CCDC reference: 1062184

Additional supporting information:  crystallographic information; 3D view; checkCIF report

## Figures and Tables

**Table 1:** Hydrogen-bond geometry (, )

*D*H*A*	*D*H	H*A*	*D* *A*	*D*H*A*
C11H11*A*O1	0.97	2.42	3.347(2)	160
C11H11*B*O1^i^	0.97	2.40	3.368(2)	172
C15H15*A*O2^ii^	0.97	2.56	3.529(2)	174
C17H17*A*O2^iii^	0.97	2.41	3.333(3)	159
C19H19*A*O3^i^	0.97	2.55	3.441(2)	152
C21H21*B*F1	0.97	2.29	3.208(4)	156

Symmetry codes: (i) 
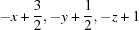
; (ii) 
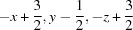
; (iii) 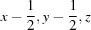.

**Table 2:** Experimental details

Crystal data	
Chemical formula	(C_16_H_36_N)_2_[Ni(C_11_H_3_F_3_N_2_O_6_)]
*M*_r_	859.78
Crystal system, space group	Monoclinic, *C*2/*c*
Temperature (K)	110
*a*, *b*, *c* ()	19.5285(3), 17.3370(3), 14.1484(3)
()	92.136(2)
*V* (^3^)	4786.83(15)
*Z*	4
Radiation type	Cu *K*
(mm^1^)	1.06
Crystal size (mm)	0.10 0.08 0.06

Data collection	
Diffractometer	Oxford Gemini S
Absorption correction	Multi-scan (*CrysAlis RED*; Oxford Diffraction, 2006 ▸)
*T*_min_, *T*_max_	0.807, 1.000
No. of measured, independent and observed [*I* > 2(*I*)] reflections	15600, 3545, 3142
*R*_int_	0.023
_max_ ()	60.5
(sin /)_max_ (^1^)	0.564

Refinement	
*R*[*F* ^2^ > 2(*F* ^2^)], *wR*(*F* ^2^), *S*	0.036, 0.102, 1.09
No. of reflections	3545
No. of parameters	277
H-atom treatment	H-atom parameters constrained
_max_, _min_ (e ^3^)	0.32, 0.20

Computer programs: *CrysAlis CCD* and *CrysAlis RED* (Oxford Diffraction, 2006 ▸), *SHELXT* (Sheldrick, 2015*a* ▸), *SHELXL2013* (Sheldrick, 2015*b* ▸), *SHELXTL* (Sheldrick, 2008 ▸), *ORTEP-3 for Windows* and *WinGX* (Farrugia, 2012 ▸), *PLATON* (Spek, 2009 ▸), *publCIF* (Westrip, 2010 ▸) and *SQUEEZE* (Spek, 2015 ▸).

## supplementary crystallographic information

### Crystal data

**Table:**

(C_16_H_36_N)_2_[Ni(C_11_H_3_F_3_N_2_O_6_)]	*F*(000) = 1856
*M**_r_* = 859.78	*D*_x_ = 1.193 Mg m^−^^3^
Monoclinic, *C*2/*c*	Cu *K*α radiation, λ = 1.54184 Å
*a* = 19.5285 (3) Å	Cell parameters from 5954 reflections
*b* = 17.3370 (3) Å	θ = 4.5–60.4°
*c* = 14.1484 (3) Å	µ = 1.06 mm^−^^1^
β = 92.136 (2)°	*T* = 110 K
*V* = 4786.83 (15) Å^3^	Block, orange
*Z* = 4	0.1 × 0.08 × 0.06 mm

### Data collection

**Table:**

Oxford Gemini S diffractometer	*R*_int_ = 0.023
ω scans	θ_max_ = 60.5°, θ_min_ = 3.4°
Absorption correction: multi-scan (*CrysAlis RED*; Oxford Diffraction, 2006)	*h* = −21→21
*T*_min_ = 0.807, *T*_max_ = 1.000	*k* = −19→19
15600 measured reflections	*l* = −15→15
3545 independent reflections	2 standard reflections every 25 reflections
3142 reflections with *I* > 2σ(*I*)	intensity decay: none

### Refinement

**Table:**

Refinement on *F*^2^	0 restraints
Least-squares matrix: full	Hydrogen site location: inferred from neighbouring sites
*R*[*F*^2^ > 2σ(*F*^2^)] = 0.036	H-atom parameters constrained
*wR*(*F*^2^) = 0.102	*w* = 1/[σ^2^(*F*_o_^2^) + (0.0577*P*)^2^ + 2.8029*P*] where *P* = (*F*_o_^2^ + 2*F*_c_^2^)/3
*S* = 1.09	(Δ/σ)_max_ = 0.001
3545 reflections	Δρ_max_ = 0.32 e Å^−^^3^
277 parameters	Δρ_min_ = −0.20 e Å^−^^3^

### Special details

**Table:**

Geometry. All e.s.d.'s (except the e.s.d. in the dihedral angle between two l.s. planes) are estimated using the full covariance matrix. The cell e.s.d.'s are taken into account individually in the estimation of e.s.d.'s in distances, angles and torsion angles; correlations between e.s.d.'s in cell parameters are only used when they are defined by crystal symmetry. An approximate (isotropic) treatment of cell e.s.d.'s is used for estimating e.s.d.'s involving l.s. planes.

### Fractional atomic coordinates and isotropic or equivalent isotropic displacement parameters (Å^2^)

**Table:**

	*x*	*y*	*z*	*U*_iso_*/*U*_eq_	Occ. (<1)
C1	0.88594 (10)	0.35368 (11)	0.65544 (14)	0.0356 (5)	
C2	0.88021 (10)	0.44362 (12)	0.65187 (14)	0.0370 (5)	
C3	0.96883 (10)	0.25942 (11)	0.72161 (15)	0.0371 (4)	
C4	0.94040 (11)	0.18979 (12)	0.69174 (16)	0.0454 (5)	
H4	0.9011	0.1893	0.6525	0.055*	
C5	0.97086 (14)	0.12088 (13)	0.72067 (18)	0.0566 (6)	
H5	0.9519	0.0743	0.7004	0.068*	0.5
C7	0.68930 (10)	0.16654 (11)	0.76809 (14)	0.0363 (4)	
H7A	0.6668	0.2105	0.7958	0.044*	
H7B	0.6729	0.1207	0.7993	0.044*	
C8	0.76537 (10)	0.17349 (11)	0.79037 (14)	0.0386 (5)	
H8A	0.7821	0.2220	0.7661	0.046*	
H8B	0.7895	0.1319	0.7600	0.046*	
C9	0.77901 (12)	0.16984 (13)	0.89696 (15)	0.0466 (5)	
H9A	0.7528	0.2100	0.9267	0.056*	
H9B	0.7631	0.1206	0.9201	0.056*	
C10	0.85411 (13)	0.17956 (14)	0.92587 (17)	0.0567 (6)	
H10A	0.8596	0.1768	0.9935	0.085*	
H10B	0.8699	0.2288	0.9045	0.085*	
H10C	0.8803	0.1393	0.8979	0.085*	
C11	0.68357 (10)	0.23650 (11)	0.61193 (14)	0.0356 (4)	
H11A	0.7321	0.2469	0.6224	0.043*	
H11B	0.6756	0.2277	0.5447	0.043*	
C12	0.64405 (11)	0.30812 (11)	0.63902 (16)	0.0412 (5)	
H12A	0.6507	0.3180	0.7062	0.049*	
H12B	0.5955	0.3006	0.6252	0.049*	
C13	0.67014 (11)	0.37655 (12)	0.58245 (17)	0.0466 (5)	
H13A	0.7196	0.3798	0.5912	0.056*	
H13B	0.6596	0.3679	0.5157	0.056*	
C14	0.63858 (16)	0.45253 (15)	0.6120 (2)	0.0789 (9)	
H14A	0.6558	0.4935	0.5739	0.118*	
H14B	0.6503	0.4623	0.6774	0.118*	
H14C	0.5897	0.4498	0.6032	0.118*	
C15	0.59008 (10)	0.14707 (11)	0.66235 (14)	0.0358 (4)	
H15A	0.5822	0.1001	0.6975	0.043*	
H15B	0.5684	0.1889	0.6956	0.043*	
C16	0.55475 (10)	0.13920 (11)	0.56594 (14)	0.0378 (5)	
H16A	0.5698	0.0922	0.5358	0.045*	
H16B	0.5668	0.1825	0.5265	0.045*	
C17	0.47760 (10)	0.13688 (13)	0.57613 (16)	0.0445 (5)	
H17A	0.4669	0.0999	0.6248	0.053*	
H17B	0.4622	0.1872	0.5966	0.053*	
C18	0.43874 (11)	0.11520 (15)	0.48520 (17)	0.0532 (6)	
H18A	0.3905	0.1147	0.4958	0.080*	
H18B	0.4529	0.0649	0.4653	0.080*	
H18C	0.4483	0.1522	0.4370	0.080*	
C19	0.70443 (10)	0.09850 (10)	0.61375 (14)	0.0348 (4)	
H19A	0.6871	0.0961	0.5487	0.042*	
H19B	0.7526	0.1121	0.6128	0.042*	
C20	0.69831 (11)	0.01936 (11)	0.65738 (15)	0.0390 (5)	
H20A	0.6509	0.0027	0.6530	0.047*	
H20B	0.7124	0.0217	0.7238	0.047*	
C21	0.74277 (12)	−0.03850 (12)	0.60717 (17)	0.0497 (6)	
H21A	0.7284	−0.0410	0.5409	0.060*	
H21B	0.7901	−0.0214	0.6111	0.060*	
C22	0.73766 (14)	−0.11826 (13)	0.65073 (17)	0.0544 (6)	
H22A	0.7661	−0.1535	0.6176	0.082*	
H22B	0.6909	−0.1356	0.6461	0.082*	
H22C	0.7527	−0.1161	0.7161	0.082*	
N1	0.94347 (8)	0.33319 (9)	0.70205 (12)	0.0356 (4)	
N2	0.66664 (8)	0.16218 (9)	0.66403 (11)	0.0338 (4)	
O1	0.84134 (7)	0.31211 (8)	0.61726 (10)	0.0410 (3)	
O2	0.92921 (7)	0.48170 (7)	0.69564 (10)	0.0391 (3)	
O3	0.83119 (7)	0.47330 (8)	0.60942 (10)	0.0445 (4)	
C6	0.9520 (2)	0.0478 (2)	0.6727 (4)	0.0499 (11)	0.5
F1	0.88379 (14)	0.04371 (15)	0.6729 (3)	0.0760 (9)	0.5
F2	0.96828 (17)	0.03869 (15)	0.5806 (2)	0.0698 (8)	0.5
F3	0.97597 (13)	−0.01539 (13)	0.7165 (2)	0.0581 (7)	0.5
Ni1	1.0000	0.41486 (2)	0.7500	0.02432 (15)	

### Atomic displacement parameters (Å^2^)

**Table:**

	*U*^11^	*U*^22^	*U*^33^	*U*^12^	*U*^13^	*U*^23^
C1	0.0395 (11)	0.0420 (11)	0.0257 (11)	0.0005 (9)	0.0080 (8)	0.0002 (8)
C2	0.0439 (11)	0.0423 (11)	0.0253 (11)	0.0022 (9)	0.0080 (9)	0.0001 (9)
C3	0.0452 (10)	0.0352 (10)	0.0312 (11)	0.0002 (8)	0.0084 (8)	0.0005 (8)
C4	0.0555 (13)	0.0409 (12)	0.0397 (13)	−0.0012 (9)	−0.0016 (10)	−0.0032 (9)
C5	0.0773 (16)	0.0352 (11)	0.0563 (15)	−0.0023 (11)	−0.0124 (12)	−0.0044 (11)
C7	0.0501 (11)	0.0329 (10)	0.0264 (11)	−0.0015 (8)	0.0098 (9)	−0.0003 (8)
C8	0.0516 (12)	0.0328 (10)	0.0318 (12)	−0.0029 (8)	0.0068 (9)	−0.0016 (8)
C9	0.0641 (14)	0.0428 (12)	0.0328 (13)	−0.0020 (10)	0.0028 (10)	0.0022 (9)
C10	0.0729 (16)	0.0570 (14)	0.0396 (14)	−0.0056 (12)	−0.0062 (11)	0.0000 (11)
C11	0.0407 (10)	0.0342 (10)	0.0322 (12)	−0.0057 (8)	0.0073 (8)	0.0031 (8)
C12	0.0496 (12)	0.0362 (11)	0.0384 (13)	−0.0024 (9)	0.0106 (9)	0.0017 (9)
C13	0.0521 (12)	0.0381 (11)	0.0505 (14)	−0.0008 (9)	0.0113 (10)	0.0080 (10)
C14	0.097 (2)	0.0411 (14)	0.101 (3)	0.0092 (14)	0.0333 (18)	0.0174 (14)
C15	0.0406 (11)	0.0334 (10)	0.0342 (12)	−0.0024 (8)	0.0120 (8)	−0.0008 (8)
C16	0.0434 (11)	0.0360 (10)	0.0348 (12)	−0.0025 (8)	0.0108 (9)	−0.0011 (8)
C17	0.0429 (11)	0.0479 (12)	0.0435 (13)	−0.0054 (9)	0.0114 (9)	0.0027 (10)
C18	0.0421 (12)	0.0666 (15)	0.0510 (15)	−0.0062 (10)	0.0040 (10)	0.0046 (11)
C19	0.0404 (10)	0.0342 (10)	0.0303 (11)	−0.0004 (8)	0.0087 (8)	−0.0038 (8)
C20	0.0500 (12)	0.0366 (10)	0.0309 (12)	−0.0019 (9)	0.0079 (9)	−0.0030 (8)
C21	0.0631 (14)	0.0442 (12)	0.0427 (14)	0.0123 (10)	0.0124 (11)	0.0008 (10)
C22	0.0783 (16)	0.0412 (12)	0.0437 (14)	0.0138 (11)	0.0028 (12)	−0.0010 (10)
N1	0.0391 (9)	0.0356 (9)	0.0324 (10)	−0.0001 (7)	0.0047 (7)	−0.0002 (7)
N2	0.0427 (9)	0.0326 (8)	0.0266 (9)	−0.0030 (7)	0.0096 (7)	−0.0009 (6)
O1	0.0432 (8)	0.0456 (8)	0.0344 (8)	−0.0040 (6)	0.0030 (6)	−0.0010 (6)
O2	0.0435 (7)	0.0365 (7)	0.0376 (8)	0.0027 (6)	0.0048 (6)	−0.0005 (6)
O3	0.0485 (8)	0.0476 (8)	0.0373 (9)	0.0078 (7)	0.0012 (7)	0.0029 (6)
C6	0.050 (3)	0.037 (2)	0.062 (3)	0.0017 (19)	0.004 (2)	−0.004 (2)
F1	0.0477 (16)	0.0465 (15)	0.134 (3)	−0.0011 (12)	0.0005 (16)	−0.0231 (17)
F2	0.100 (2)	0.0521 (16)	0.0572 (19)	0.0026 (15)	−0.0005 (16)	−0.0133 (14)
F3	0.0630 (17)	0.0351 (13)	0.076 (2)	0.0013 (11)	0.0042 (12)	0.0005 (12)
Ni1	0.0293 (2)	0.0226 (2)	0.0215 (3)	0.000	0.00539 (16)	0.000

### Geometric parameters (Å, º)

**Table:**

C1—O1	1.239 (2)	C14—H14B	0.9600
C1—N1	1.330 (3)	C14—H14C	0.9600
C1—C2	1.564 (3)	C15—C16	1.512 (3)
C2—O3	1.224 (2)	C15—N2	1.517 (2)
C2—O2	1.300 (2)	C15—H15A	0.9700
C3—C4	1.388 (3)	C15—H15B	0.9700
C3—N1	1.396 (2)	C16—C17	1.519 (3)
C3—C3^i^	1.433 (4)	C16—H16A	0.9700
C4—C5	1.389 (3)	C16—H16B	0.9700
C4—H4	0.9300	C17—C18	1.516 (3)
C5—C5^i^	1.383 (5)	C17—H17A	0.9700
C5—H5	0.9300	C17—H17B	0.9700
C7—C8	1.512 (3)	C18—H18A	0.9600
C7—N2	1.523 (2)	C18—H18B	0.9600
C7—H7A	0.9700	C18—H18C	0.9600
C7—H7B	0.9700	C19—C20	1.511 (3)
C8—C9	1.523 (3)	C19—N2	1.519 (2)
C8—H8A	0.9700	C19—H19A	0.9700
C8—H8B	0.9700	C19—H19B	0.9700
C9—C10	1.517 (3)	C20—C21	1.520 (3)
C9—H9A	0.9700	C20—H20A	0.9700
C9—H9B	0.9700	C20—H20B	0.9700
C10—H10A	0.9600	C21—C22	1.519 (3)
C10—H10B	0.9600	C21—H21A	0.9700
C10—H10C	0.9600	C21—H21B	0.9700
C11—C12	1.519 (3)	C22—H22A	0.9600
C11—N2	1.527 (2)	C22—H22B	0.9600
C11—H11A	0.9700	C22—H22C	0.9600
C11—H11B	0.9700	N1—Ni1	1.9047 (16)
C12—C13	1.529 (3)	O2—Ni1	1.9407 (13)
C12—H12A	0.9700	C6—F1	1.333 (5)
C12—H12B	0.9700	C6—F3	1.335 (5)
C13—C14	1.519 (3)	C6—F2	1.361 (6)
C13—H13A	0.9700	F3—F3^i^	1.308 (5)
C13—H13B	0.9700	Ni1—N1^i^	1.9047 (16)
C14—H14A	0.9600	Ni1—O2^i^	1.9408 (13)
			
O1—C1—N1	128.92 (18)	C16—C15—H15B	108.2
O1—C1—C2	121.17 (17)	N2—C15—H15B	108.2
N1—C1—C2	109.91 (16)	H15A—C15—H15B	107.3
O3—C2—O2	124.63 (18)	C15—C16—C17	109.73 (16)
O3—C2—C1	119.25 (18)	C15—C16—H16A	109.7
O2—C2—C1	116.12 (16)	C17—C16—H16A	109.7
C4—C3—N1	127.00 (19)	C15—C16—H16B	109.7
C4—C3—C3^i^	119.50 (12)	C17—C16—H16B	109.7
N1—C3—C3^i^	113.51 (11)	H16A—C16—H16B	108.2
C3—C4—C5	119.7 (2)	C18—C17—C16	113.12 (18)
C3—C4—H4	120.1	C18—C17—H17A	109.0
C5—C4—H4	120.1	C16—C17—H17A	109.0
C5^i^—C5—C4	120.69 (13)	C18—C17—H17B	109.0
C5^i^—C5—H5	119.7	C16—C17—H17B	109.0
C4—C5—H5	119.7	H17A—C17—H17B	107.8
C8—C7—N2	116.96 (15)	C17—C18—H18A	109.5
C8—C7—H7A	108.1	C17—C18—H18B	109.5
N2—C7—H7A	108.1	H18A—C18—H18B	109.5
C8—C7—H7B	108.1	C17—C18—H18C	109.5
N2—C7—H7B	108.1	H18A—C18—H18C	109.5
H7A—C7—H7B	107.3	H18B—C18—H18C	109.5
C7—C8—C9	109.72 (16)	C20—C19—N2	114.92 (16)
C7—C8—H8A	109.7	C20—C19—H19A	108.5
C9—C8—H8A	109.7	N2—C19—H19A	108.5
C7—C8—H8B	109.7	C20—C19—H19B	108.5
C9—C8—H8B	109.7	N2—C19—H19B	108.5
H8A—C8—H8B	108.2	H19A—C19—H19B	107.5
C10—C9—C8	113.21 (18)	C19—C20—C21	110.68 (17)
C10—C9—H9A	108.9	C19—C20—H20A	109.5
C8—C9—H9A	108.9	C21—C20—H20A	109.5
C10—C9—H9B	108.9	C19—C20—H20B	109.5
C8—C9—H9B	108.9	C21—C20—H20B	109.5
H9A—C9—H9B	107.8	H20A—C20—H20B	108.1
C9—C10—H10A	109.5	C22—C21—C20	111.33 (18)
C9—C10—H10B	109.5	C22—C21—H21A	109.4
H10A—C10—H10B	109.5	C20—C21—H21A	109.4
C9—C10—H10C	109.5	C22—C21—H21B	109.4
H10A—C10—H10C	109.5	C20—C21—H21B	109.4
H10B—C10—H10C	109.5	H21A—C21—H21B	108.0
C12—C11—N2	116.55 (16)	C21—C22—H22A	109.5
C12—C11—H11A	108.2	C21—C22—H22B	109.5
N2—C11—H11A	108.2	H22A—C22—H22B	109.5
C12—C11—H11B	108.2	C21—C22—H22C	109.5
N2—C11—H11B	108.2	H22A—C22—H22C	109.5
H11A—C11—H11B	107.3	H22B—C22—H22C	109.5
C11—C12—C13	108.66 (17)	C1—N1—C3	129.06 (17)
C11—C12—H12A	110.0	C1—N1—Ni1	116.48 (13)
C13—C12—H12A	110.0	C3—N1—Ni1	114.46 (13)
C11—C12—H12B	110.0	C15—N2—C19	111.27 (14)
C13—C12—H12B	110.0	C15—N2—C7	105.92 (14)
H12A—C12—H12B	108.3	C19—N2—C7	111.10 (14)
C14—C13—C12	112.47 (19)	C15—N2—C11	111.66 (14)
C14—C13—H13A	109.1	C19—N2—C11	105.63 (14)
C12—C13—H13A	109.1	C7—N2—C11	111.37 (14)
C14—C13—H13B	109.1	C2—O2—Ni1	112.72 (12)
C12—C13—H13B	109.1	F1—C6—F3	106.8 (4)
H13A—C13—H13B	107.8	F1—C6—F2	105.4 (4)
C13—C14—H14A	109.5	F3—C6—F2	105.0 (4)
C13—C14—H14B	109.5	F3^i^—F3—C6	124.5 (2)
H14A—C14—H14B	109.5	N1^i^—Ni1—N1	83.96 (9)
C13—C14—H14C	109.5	N1^i^—Ni1—O2	168.49 (6)
H14A—C14—H14C	109.5	N1—Ni1—O2	84.71 (6)
H14B—C14—H14C	109.5	N1^i^—Ni1—O2^i^	84.72 (6)
C16—C15—N2	116.49 (15)	N1—Ni1—O2^i^	168.49 (6)
C16—C15—H15A	108.2	O2—Ni1—O2^i^	106.67 (8)
N2—C15—H15A	108.2		
			
O1—C1—C2—O3	−1.0 (3)	C3^i^—C3—N1—C1	−176.8 (2)
N1—C1—C2—O3	178.08 (18)	C4—C3—N1—Ni1	−176.64 (18)
O1—C1—C2—O2	178.84 (17)	C3^i^—C3—N1—Ni1	3.0 (3)
N1—C1—C2—O2	−2.0 (2)	C16—C15—N2—C19	−58.4 (2)
N1—C3—C4—C5	−177.5 (2)	C16—C15—N2—C7	−179.28 (16)
C3^i^—C3—C4—C5	2.9 (4)	C16—C15—N2—C11	59.3 (2)
C3—C4—C5—C5^i^	0.3 (5)	C20—C19—N2—C15	−62.1 (2)
N2—C7—C8—C9	−174.95 (15)	C20—C19—N2—C7	55.7 (2)
C7—C8—C9—C10	−177.74 (18)	C20—C19—N2—C11	176.60 (16)
N2—C11—C12—C13	177.74 (17)	C8—C7—N2—C15	174.15 (16)
C11—C12—C13—C14	−174.2 (2)	C8—C7—N2—C19	53.2 (2)
N2—C15—C16—C17	−171.21 (16)	C8—C7—N2—C11	−64.3 (2)
C15—C16—C17—C18	−169.98 (18)	C12—C11—N2—C15	50.4 (2)
N2—C19—C20—C21	−175.07 (17)	C12—C11—N2—C19	171.52 (17)
C19—C20—C21—C22	179.53 (19)	C12—C11—N2—C7	−67.8 (2)
O1—C1—N1—C3	−0.8 (3)	O3—C2—O2—Ni1	−177.48 (16)
C2—C1—N1—C3	−179.87 (18)	C1—C2—O2—Ni1	2.7 (2)
O1—C1—N1—Ni1	179.39 (16)	F1—C6—F3—F3^i^	−131.6 (5)
C2—C1—N1—Ni1	0.4 (2)	F2—C6—F3—F3^i^	116.8 (5)
C4—C3—N1—C1	3.6 (3)		

Symmetry code: (i) −*x*+2, *y*, −*z*+3/2.

### Hydrogen-bond geometry (Å, º)

**Table:**

*D*—H···*A*	*D*—H	H···*A*	*D*···*A*	*D*—H···*A*
C11—H11*A*···O1	0.97	2.42	3.347 (2)	160
C11—H11*B*···O1^ii^	0.97	2.40	3.368 (2)	172
C15—H15*A*···O2^iii^	0.97	2.56	3.529 (2)	174
C17—H17*A*···O2^iv^	0.97	2.41	3.333 (3)	159
C19—H19*A*···O3^ii^	0.97	2.55	3.441 (2)	152
C21—H21*B*···F1	0.97	2.29	3.208 (4)	156

Symmetry codes: (ii) −*x*+3/2, −*y*+1/2, −*z*+1; (iii) −*x*+3/2, *y*−1/2, −*z*+3/2; (iv) *x*−1/2, *y*−1/2, *z*.
